# Beyond life cycle thinking: A perspective

**DOI:** 10.1007/s13280-025-02282-x

**Published:** 2025-10-26

**Authors:** Hannes Geist, Frank Balle

**Affiliations:** 1 Walter-and-Ingeborg-Herrmann Chair for Power Ultrasonics and Engineering of Functional Materials (EFM), Department of Sustainable Systems Engineering (INATECH), Faculty of Engineering, University of Freiburg, Emmy Noether Str. 2, 79110 Freiburg i. Br., Germany; 2 Freiburg Materials Research Center FMF, Stefan-Meier Str. 21, 79104 Freiburg i. Br., Germany; 3 Fraunhofer-Institute for High-Speed Dynamics, Ernst Mach Institute (EMI), Ernst-Zermelo Str. 4, 79104 Freiburg i. Br., Germany

**Keywords:** Circular economy, End of life, Life cycle, Life cycle thinking, Sustainability, Systems thinking

## Abstract

Life cycle thinking is a fundamental concept of sustainability endeavors in most disciplines. The share of non-expert users applying life cycle thinking-based methodologies and tools is nowadays already significantly larger than the respective expert community. This perspective discusses life cycle thinking with a focus on its implications, limitations, and potential ways to overcome them from this non-expert perspective. While building on a long history and bringing undoubted advantages, we raise the question of whether thinking in life cycles alone is sufficient in light of today’s sustainability challenges. Four key limitations are found in the literature, all directly related to the definition of the concept itself. Solution attempts and sensitivity for these issues exist in the expert community only. We raise the discussion about going beyond the often-eponymous focus on life cycle thinking with holistic systems thinking and updated terminology.

## Introduction to life cycle thinking and its application

Thinking in life cycles of product systems is a fundamental paradigm of environmental life cycle assessment (E-LCA) (ISO 2021), social life cycle assessment (S-LCA) (UNEP 2020; ISO 2024a), life cycle costing (LCC) (SETAC 2011), life cycle sustainability assessment (LCSA) (UNEP et al. 2011; Valdivia Mercado and Sonnemann 2024), eco design (Rossi et al. 2016), circular design (Hollander et al. 2017), sustainable design (UNEP and TU Delft 2009), life cycle engineering (Hauschild et al. 2017), and sustainable engineering (Allen and Shonnard 2012; Bakshi 2019; Mulligan 2020) among others. The purpose of life cycle thinking is to prevent relocating burden to other life cycle phases or processes by overcoming the incomplete and misguiding focus on single life cycle stages (ISO 2024a; Strothmann and Fava 2024). The origin of the concept dates back to the late 1960s for environmental assessments (Guinée et al. 2011) and to the 1950s for life cycle costing (SETAC 2011). Since then, it has emerged as a foundational concept to address most sustainability challenges (Strothmann and Fava 2024). Its main application areas are in product assessment (E-LCA, S-LCA, LCC, LCSA) and optimization or management (eco design, circular design, sustainable design, life cycle engineering, sustainable engineering).

Life cycle thinking and its related concepts are internationally standardized. Life cycle thinking, as defined in ISO 14050 (ISO 2020) is based on the concepts of “product”, “product system”, and “life cycle”. The life cycle is defined as the “consecutive interlinked stages (of a product system [only in ISO 14040]) from raw material acquisition or generation from natural resources to final disposal” (ISO 2020, 2021). A product is defined widely as “any goods or service” (ISO 2020, 2021). The definition of product system applies the life cycle concept to products, as a “collection of unit processes […] which models the life cycle of a product” (ISO 2021). Life cycle thinking, in the context of environmental management, is therefore the “consideration of the environmental aspects relating to a product during its entire life cycle” (ISO 2020). In the context of the circular economy, life cycle thinking is about the consideration of circularity aspects, and for sustainability assessments, about considering sustainability aspects during the entire life cycle (ISO 2024b).

Since the establishment of the paradigm of life cycle thinking, the urgency of sustainability problems has increased, and a better understanding of the systemic nature of the complex causes of sustainability problems has evolved. Furthermore, non-experts increasingly use life cycle thinking and the methodologies and tools based on it. This is true for the application in industry and all academic disciplines that are not industrial ecology. For example, the guidance document on sustainability for the engineering profession of the UK engineering council requires engineers even to “adopt life cycle assessment as normal practice” (Engineering Council 2021). Increasing application by non-experts is undoubtedly desirable to gain momentum toward sustainability transformations. At the same time, it brings new conceptual and methodological challenges. In this perspective, we discuss key limitations of life cycle thinking and potential pathways to overcome them. The perspective focuses on our own non-expert but engineering perspective in light of today’s urgency and understanding of sustainability problems.

While life cycle thinking perfectly fulfills its’ initial purpose stated above, this perspective sheds light on what is missed out when focusing narrowly on life cycle thinking as typical for non-experts; or in other words highlights what lies “beyond life cycle thinking”. As non-experts, we frame professionals and students applying life cycle thinking in their work, who have enjoyed introductory courses at universities or in professional education, and are familiar with the basic literature of the field. Conversely, experts are seen as highly educated in life cycle thinking-based concepts, methods, and tools, and build their careers primarily around applying and further developing them. Existing reviews and critiques, many of which are built upon in this work, focus on specific types of life cycle assessment, optimization, or management methods. No literature has been found discussing limitations of life cycle thinking in general. In the following, we summarize such limitations and their implications especially for non-experts, recent scientific advancements providing methodological options for how to overcome them, and finally the research and changes in practice that are needed to resolve the listed issues in the future.

## Limitations of life cycle thinking

Limitations are clustered as four issues. The listed limitations are inherent to the terminology used and reflected in the simplified tools available to and daily practice of non-expert users. Examples for such streamlined assessment and optimization tools based on life cycle thinking that address primarily non-experts are the Eco Audit tool developed by Mike Ashby (Ashby 2021), used in teaching engineering students at hundreds of universities worldwide with Granta EduPack (ANSYS Granta 2025a) and available to businesses with Granta Selector from Ansys (ANSYS Granta 2025b), the product carbon tool by One Click LCA that is compatible with many Autodesk tools (One Click LCA 2025), the streamlined LCA tool Solidworks Sustainability (Solidworks 2025), or the LCA Calculator by Sphera (Sphera 2024) to name just a few. A comprehensive summary of such tools is provided by Pollini and Rognoli (Pollini and Rognoli 2021).

Solution attempts for all listed limitations exist in the expert community and are summarized below, but in parallel to and not as a part of life cycle thinking. These solution attempts barely find their way into non-expert practice since they would require awareness that they exist, in-depth methodological know-how, integration in tools that address non-experts, and significant additional efforts, going beyond the “obvious” name-giving implications of life cycle thinking as discussed in detail in section "Implications and outlook".

While being a reasonable analogy chosen historically for overcoming the focus on single processes or life cycle phases, the term “life” in terms like product life, life cycle, or end of life faces an etymological problem. The term implies a few aspects generally associated with literal life, causing significant conceptual problems, especially in the reality of non-expert users. The concept of life implies inevitable death, leading to "The longitudinal issue". Life also typically refers to individuals, causing "The ‘individual’ issue", which causes "The transversal issue"*,* and "The vertical issue".

### The longitudinal issue

Death is perceived as reversible in only a few cultures (reincarnation). The focus of life cycle thinking is therefore almost always on one single life cycle ending with an end-of-life stage, called “grave”. But in the systems typically tackled by life cycle thinking in reality, obsolescence (“death”) can be postponed, reversed, or even designed out (Hollander et al. 2017). That way, the inherently linear analogy of life hinders thinking and progress toward a circular economy (Braungart et al. 2007). Vice versa, it stabilizes today’s linear economy, only allowing for incremental rather than transformational and systemic sustainability improvements.

One might argue that a single life cycle can include several use cycles, even with today’s terminology. Such use cycles are separated by an end of use, differentiating from an end of life through lifetime extension measures for products (IRP 2018). This includes all standard value-retention processes like reuse, repair, refurbishment, and remanufacturing. The wording implies that even with multiple use cycles resulting from life extension, still the life of one “individual” product is investigated. In the case of repair and reuse, this line of argumentation works. But during refurbishment and remanufacturing, significant changes can be made to the product’s specification (~ identity), for instance, through upgrades (IRP 2018). In remanufacturing, parts from several used products across types and generations can be mixed with new parts to produce new products (in Europe, they are even legally considered new products) (European Commission 2024). A remanufactured product has its own specifications and product identity. It must be introduced to the market newly, fulfilling the exact regulatory requirements that all linearly produced new products must follow (DIN SPEC 91472:2023-06 2023). It is, therefore, no longer related to any initial product, making the differentiation between use cycles and life cycles, at least from this point on, blurry. If life cycle thinking is applied for assessments of circular products or comparisons of circular with linear products, a system boundary covering only one life or use cycle can still be useful, e.g., for benchmarking (Peng et al. 2022). Yet, optimizing the circular design of products or systems requires widening the system boundary to include all use and life cycles relevant to the optimization task.

There are attempts in the expert community to overcome the “literal” single life cycle system boundary or even perform multiple use cycle assessments or optimizations, tackling the longitudinal problem (Suhariyanto et al. 2017; Asif et al. 2021; van Stijn et al. 2021; Larsen et al. 2022). Secondary functions from by-products or recycled materials entering second life cycles are typically considered in many assessment methods. Yet, all simplified methods and tools to support non-expert users reviewed, for instance in life cycle engineering (Hauschild et al. 2017) or eco-audits in materials selection (Ashby 2021) are limited to single product life cycles, don’t allow to model multiple use cycle systems, and offer circular strategies only as alternative end-of-life pathways if at all.

### The “individual” issue

Living organisms are perceived as individuals; manufactured goods and services are not. A life cycle literally always refers to one specific living individuum. The common abstraction of this concept in life cycle thinking is the investigation of one average individual product system entering into “life” at a certain point in time, or as stated by the definitions, “a product” (ISO 2020, 2021). There are a handful of methodological issues with that. One specific type of product or product service system, produced or sold for 10 years, has a significantly different life cycle impact if produced in the first year compared to the last year, even if the technical specifications and final services provided remain constant. This is due to the regionally specific evolution of the foreground and background system over time, demanding time-dependent impact characterization (Lueddeckens et al. 2020). For example the carbon emissions intensity of electricity generation has dropped globally by 12% in the ten years from 2015 until 2024, but has almost halved in the European Union during the same timespan (IEA 2025). Prospective analysis approaches provide solutions, but remain challenging, even for the expert community (Moni et al. 2020). The analogy “life” narrows the scope down to the immediate storyline of a specific “life cycle”, but its systemic context, influence on, effects in, and feedback from it, are not implied. This starts with the interrelation of product systems with business models. While being a big own field of research, it typically does not enter methods and tools that turn life cycle thinking into action in fields like engineering or materials science (Allen and Shonnard 2012; Hauschild et al. 2017; Ashby 2021). That way, significant sustainability potential (Sarasini et al. 2024) and risks (Castro et al. 2022) are out of sight. A review of the climate impacts of product service systems shows, for example, that sharing business models can lead to emission reductions of up to 80%, while also posing risks of emission increases of up to 30% (Sarasini et al. 2024). Further, the interrelation with the bigger socio-technological system in which the system under investigation is embedded is literally out of focus. That way, rebound effects are out of scope for a “life cycle” since they evolve outside the immediate objects’ “life” under investigation. Considering that around half of all sustainability potential never gets realized because of rebounds, with even backfire detected in many studies, this is a major shortcoming (Andrew and Pigosso 2024). Consequential life cycle assessment provides a mature solution for that issue (Earles and Halog 2011), but due to its complexity, primarily for the expert community, and due to its detailed data requirements, mostly for ex post assessments and not for ex ante optimizations. The focus on average product life cycles is useful for single use and life cycles. Highly heterogeneous usage conditions and behavior make “a product” not sufficiently precise for multiple use or life cycle systems. Heterogeneous post-usage conditions of products, components, and materials with respect to contamination, deficits, and defects (Geist and Balle 2024b) result in different numbers of possible use or life cycles, necessary processing steps during value-retention processes like remanufacturing, and related efforts (Geist and Balle 2024a). Life concepts concerning individual products also do not call for any further sub-differentiation of products into a product, component, and material level. If the products’ life cycle is over, then that defines the system boundary. Typically, substantial timely mismatches exist between these layers’ “lifetimes”. Washing machines, for example, are reported to typically fail and being discarded after 6 years due to wear of inaccessible bearings, while 70 wt% of the components a washing machines is composed of would allow for at least 18 years of usage (Cooper et al. 2014). Such a sub-differentiation is key to enabling material efficiency or value-retention processes like remanufacturing and refurbishment in a circular economy. A first definition tackling this last issue is provided by ISO 59014:2024 (ISO 2024b).

### The transversal issue

A direct result of the “individual issue” with the focus on “a product” described in the previous section is focusing on individual product systems without considering their interrelation with the product portfolio they are a part of. While some kinds of sustainability optimizations can be performed product by product individually, others like positive lists for materials selection, sustainable sourcing, material diversity reductions, or platform design to name just a few, follow the scope of product groups, the full product portfolio of a company, a market, or even bigger scopes (Braungart et al. 2007; McAloone and Pigosso 2021). This also imposes an inaccuracy for assessment since potential rebounds in the product portfolio or the market would be out of focus (Lange et al. 2021). While life cycle engineering recently expanded its conceptual scope to consider the transversal issue, the related methods and tools do not (Hauschild et al. 2017). Consequential modeling allows overcoming the transversal issue methodologically (Earles and Halog 2011), but requires significant expertise.

### The vertical issue

Another result of the “individual issue” is the terminology-inherent ignorance of scale. If one average “individuum” is investigated (“a product” (ISO 2020, 2021)), its’ volume or total number is out of scope. But sustainability is very much about scale. Firstly, when investigating individuals rather than systems at scale, some issues that can be ignored for single products make a tremendous difference at scale. The few grams of tire abrasion in the life cycle of a car, the abrasion of paint, or plastic products ending up in the environment might be cut off, or at least get no attention in the mass-balance of a typical life cycle inventory. Considering the more than 1.5 billion cars globally, all applications of paint worldwide, and all plastic products ending up in the environment, these few grams per individual system sum up to be the source for most of all microplastics (Thompson et al. 2024). Secondly, the urgency and severity of sustainability problems make potential sustainability solutions a question of scaling times. Potential solutions that cannot scale fast enough compared to the problems’ urgency and magnitude cannot make a significant difference (Allwood 2018). This key question for sustainability transformations is not inherent in life cycle thinking as defined by ISO. Thirdly, there is increasing agreement about the existence of absolute sustainability limits, including ecological upper limits and social lower limits defining a “safe and just operating space for humanity” (Rockström et al. 2009; Raworth 2012, 2017; Richardson et al. 2023). This requires widening the scope of life cycle considerations in a systemic way and connecting traditional bottom-up perspectives to absolute top-down sustainability limits with the concept of scale (Hauschild et al. 2017, 2020). While methodological solution attempts exist with prospective modeling (Moni et al. 2020) and absolute sustainability assessment (Kosnik et al. 2022), these are out of notice for the non-expert community and not inherent to life cycle thinking.

## Implications and outlook

**Fig. 1:**
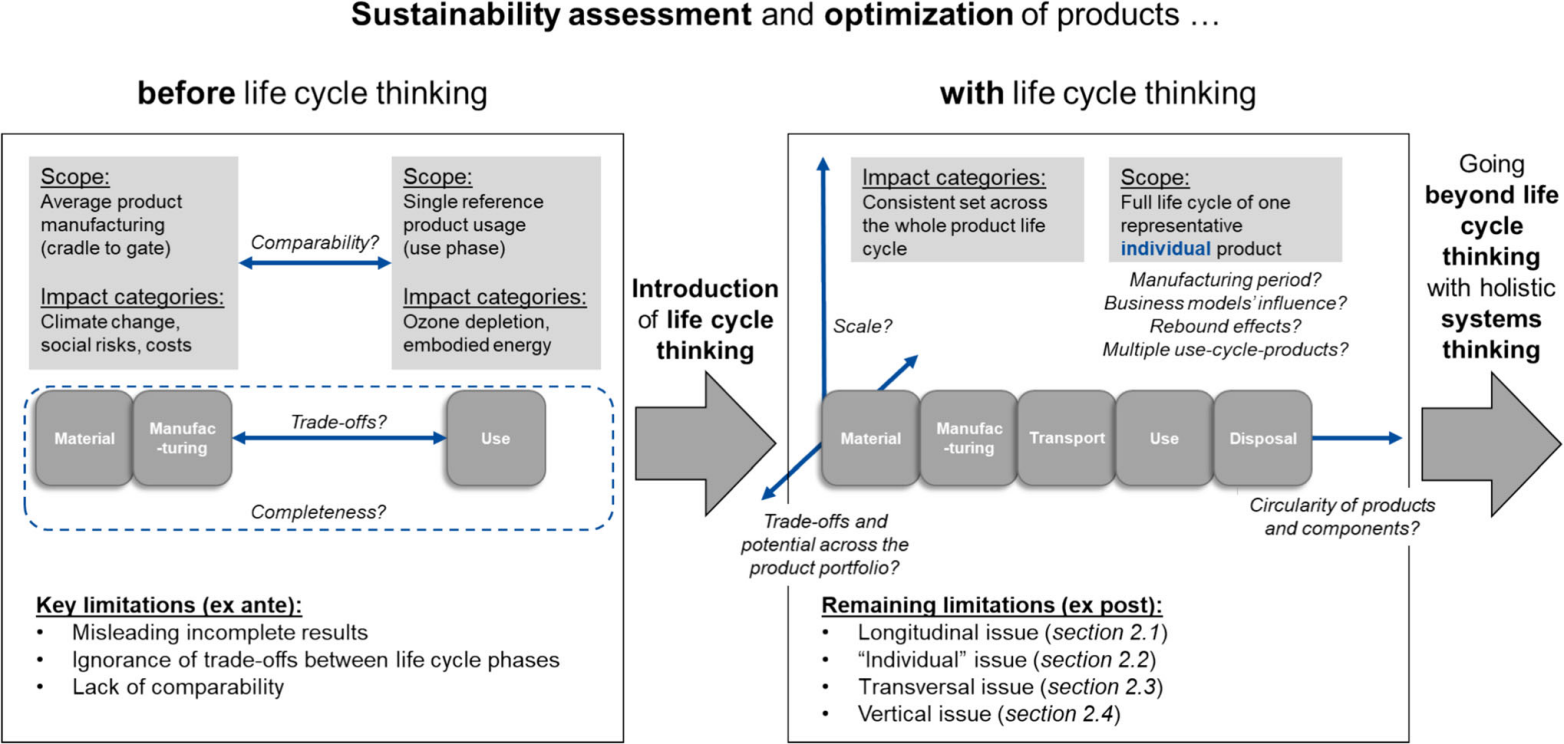
Graphical summary of the benefits of and remaining limitations after introducing life cycle thinking to product-focused sustainability assessment and optimizations, especially relevant to non-experts

The benefits provided by the implementation of life cycle thinking (section "Introduction to life cycle thinking and its application") and the remaining limitations of a too narrow focus on life cycle thinking exclusively (section "Limitations of life cycle thinking") are summarized in Fig. 1.

No literature, methods, or tools for non-experts where found, that addresses even one of the four key limitations of life cycle thinking, summarized in the previous section. Even introductory texts for the expert community frequently miss to mention the concepts’ limitations and their implications (Strothmann and Fava 2024). Basing attempts toward sustainability on the paradigm of life cycle thinking in its’ ISO-defined sense exclusively seems overall problematic. The analogy is useful and effective for overcoming the focus on single life cycle phases. Nevertheless, defining this focus unintentionally sets several important systemic sustainability considerations outside of this focus. This is of critical relevance, since life cycle thinking is eponymous for most of the methodologies and concepts listed at the beginning of this work (e.g., *life cycle* assessment or *life cycle* engineering). It does not mean that there are no methodological options for tackling the issues that are out of this defined focus, as illustrated in section "Limitations of life cycle thinking". It only means they are not part of life cycle thinking based on its ISO-standardized, clearly defined meaning. Solution attempts exist in the shadow of the conceptually dominant and eponymous life cycle thinking, barely visible to the non-expert community. Some conceptual problems summarized in the previous section pose methodological challenges even to the expert community on life cycle thinking-based methodologies of all kinds, are not noticed by all researchers and even further away from being generally resolved for assessment, optimization, and design. If even experts are challenged, how can the sustainability contribution of all the researchers and practitioners who are just applying (not questioning) life cycle thinking to their work not be significantly limited by the listed problems? With this perspective, we want to sensitize experts to also consider the non-experts’ point of view, to be ultimately effective together. Imagine being an industry professional or an engineering researcher, starting to use typically simplified/streamlined life cycle thinking-based assessment, design, or management methods or tools, and you have little time to familiarize yourself with its theoretical background. Then wording, definitions, and resulting implications fundamentally shape your understanding of what you should consider and what not. This will unavoidably be reflected in your practice.

While the purpose of this work is to discuss the conceptual status quo with a focus on its limitations and methodological options to tackle them, it is not to deliver an ultimate solution for the issues sketched. It is also not a holistic analysis of challenges with life cycle-based methods in general or for the non-expert community specifically, which would have to go far beyond the issues sketched here (e.g., false certainty through quantification with little transparency about the high uncertainties (Kuczenski 2019), or the transparency and quality of the data they rely on (Guo et al. 2025)). Still, there are generic ways for the field to move forward, besides the complex methodological options for going beyond life cycle thinking, summarized in section "Limitations of life cycle thinking".

Systems thinking as one way to handle complexity, that cannot be effectively addressed with reductionistic and mechanistic ways of thinking, has a long tradition in several different disciplines. This complexity can be independent of sustainability challenges and refer to issues like the increasing complexity that engineers face with understanding the technical and contextual dimensions of their work (Dugan et al. 2022) or that management faces with understanding socio-economic systems (Jackson 2009). These specific existing disciplinary practices typically adopt selected useful parts of general systems thinking theory, while modifying others, making them distinguishable types of systems thinking (Frank 2000; Jackson 2009).

Complementary, holistic systems’ thinking is seen as a pre-condition for transformational progress toward sustainability (Voulvoulis et al. 2022). It is widely agreed upon as a key competency in sustainability, specifically for problem analyses as a base for the development of effective sustainability transition strategies (Wiek et al. 2011; Brundiers et al. 2021; Redman and Wiek 2021). Both are main steps of transformational sustainability research (Wiek and Lang 2016) and correspond to the main application areas of life cycle thinking, assessment, and optimization. Systems’ thinking is additionally mentioned to be an important methodological way, for overcoming limitations of life cycle thinking-based methods (Onat et al. 2017).

Building upon the diverse sustainability independent systems’ thinking practices across disciplines can be an effective way to expand into systems thinking in the context of sustainability, as increasingly fostered in higher education. Making this connection when introducing non-experts to life cycle thinking-based methods or tools and categorizing life cycle thinking as one specific scope of systems thinking, can unveil the “four issues” naturally to them. With this way of framing, the further development of streamlined life cycle thinking-based assessment and optimization methods and tools for non-experts in light of the sketched issues is an important research need.

Beyond that, alternative nomenclature might serve as a potential quick fix. Which of the four issues, or new ones, still come to your mind when reading the following statements:

- Multiple use cycle product service systems in their bigger socio-economic-technical context at their actual real-world scale;
- Systems thinking around fulfilling needs and wants;
- Systemic use cycle thinking, considering the system, product (product use cycles), component (component use cycles), and material level (material use cycles) at scale and time.

## Data Availability

Data sharing is not applicable to this article as no datasets were generated or analyzed during the current study.
